# Culture-independent approaches to chlamydial genomics

**DOI:** 10.1099/mgen.0.000145

**Published:** 2018-01-03

**Authors:** Alyce Taylor-Brown, Danielle Madden, Adam Polkinghorne

**Affiliations:** Centre for Animal Health Innovation, Faculty of Science, Health, Education and Engineering, University of the Sunshine Coast, Sippy Downs, Australia

**Keywords:** *Chlamydiae*, culture-independent, metagenomics, diversity, novel species

## Abstract

The expanding field of bacterial genomics has revolutionized our understanding of microbial diversity, biology and phylogeny. For most species, DNA extracted from culture material is used as the template for genome sequencing; however, the majority of microbes are actually uncultivable, and others, such as obligate intracellular bacteria, require laborious tissue culture to yield sufficient genomic material for sequencing. *Chlamydiae* are one such group of obligate intracellular microbes whose characterization has been hampered by this requirement. To circumvent these challenges, researchers have developed culture-independent sample preparation methods that can be applied to the sample directly or to genomic material extracted from the sample. These methods, which encompass both targeted [immunomagnetic separation-multiple displacement amplification (IMS-MDA) and sequence capture] and non-targeted approaches (host methylated DNA depletion-microbial DNA enrichment and cell-sorting-MDA), have been applied to a range of clinical and environmental samples to generate whole genomes of novel chlamydial species and strains. This review aims to provide an overview of the application, advantages and limitations of these targeted and non-targeted approaches in the chlamydial context. The methods discussed also have broad application to other obligate intracellular bacteria or clinical and environmental samples.

**Impact Statement**

Until recently, our understanding of the diversity of the bacterial kingdom has been largely limited by a reliance on the use of technically challenging and time-consuming methods whereby bacteria are cultured on media or in host cells. However, the recent advancement of culture-independent methods combined with next-generation sequencing has revolutionized our ability to study microbial biology, diversity and evolution. In particular, the expanding application of these techniques has greatly advanced our understanding of the biology and genomic diversity of the obligate intracellular bacterial phylum *Chlamydiae.* Here, we have summarized six key culture-independent methods for chlamydial genomics. These methods can be used on human, veterinary and environmental samples to select for chlamydial DNA prior to genome sequencing and analysis. This review may be used as a guideline for the selection of appropriate culture-independent technique(s) depending largely on sample type. We suggest that targeted genome capture methods should be applied if the pathogen of interest is known and a reference genome is available. However, if the pathogen is unknown, and novel species discovery is the aim, non-targeted (meta)genome capture methods should be used. The methods discussed in this review also have broad application to other microorganisms or clinical and environmental samples.

## Introduction

### From the cultivable minority to metagenomes to microbial genomes

Microbial community profiling and ecology analysis has proven to be a useful tool for discovering diverse, novel microbial taxa in the far reaches of our biosphere. While initial microbial diversity studies involved culture-dependent methods [1], the development and use of rRNA-based molecular methods [2, 3] led to the understanding that cultivable bacteria only represent ~1 % of the number of bacterial species in a given sample [4–6]. As such, microbial profiling tools quickly switched to culture-independent molecular methods, commonly using conserved regions of the 16S rRNA gene to characterize microbial diversity in environments such as soil, sediment and water, as well as human gut, skin and oral microbiomes [4–9]. In more recent years, there has been another shift toward deep metagenomic sequencing, in which the entire DNA extract is subject to shotgun sequencing [10–12], and single-cell genomics, whereby the genome of a single bacterium is sequenced exclusive of the background of its community [13–18]. Both methods allow for the characterization of distinct microbial genomes.

Besides a greater understanding of microbial diversity, the introduction and subsequent extensive use of microbial genomics has implications for food safety [19], antimicrobial resistance [20], drug development [21] and disease epidemiology [22, 23]. Microbial genomics is an ever-expanding field, yet particular groups of bacteria are still challenging to sequence, such as obligate intracellular bacteria that require labour-intensive tissue culture and semi-purification away from the host cells. Helping to address these challenges are novel depletion or enrichment methods that target certain components of the sample [24–28], and are described in this review with reference to *Chlamydiae*.

Members of the phylum *Chlamydiae* are globally significant, widely distributed human and animal pathogens. *Chlamydia trachomatis* remains the cause of the most common bacterial sexually transmitted infection worldwide, as well as the leading cause of preventable blindness [29]. Several more distantly related taxa such as *Waddlia chondrophila* have emerged as species of human and veterinary importance [30]. Furthermore, the extent to which chlamydiae inhabit various ecological niches is still being unravelled [31, 32].

Genomics studies are proving key to the ongoing characterization of chlamydial diversity and pathogenicity, yet the obligate host-association and low abundance of chlamydiae in many environmental and clinical samples has presented a challenge for such studies. This review focuses on methods that may be applied to overcome some of these challenges.

## The importance of chlamydial genomics studies for understanding chlamydial biology

*Chlamydiae* are obligate intracellular bacteria, characterized by a specialized biphasic life cycle that alternates between an infectious elementary body (EB) and vegetative reticulate body (RB) that requires growth within the cytoplasm of the host cell [33]. This requirement has hindered attempts to understand the biology of chlamydial species and thus served as a significant barrier for the characterization of novel chlamydial pathogens. The application of genome sequencing to chlamydiae revolutionized our understanding of chlamydial biology, especially because genetic manipulation systems were lacking, and are still in their infancy [34, 35], transforming the way that we think about these important intracellular pathogens.

A key example of such insight has been the discovery through genomics that chlamydiae encode complete recombination enzyme pathways facilitating homologous recombination between strains despite a purported bottleneck associated with their growth restriction to an intracellular vacuole [36–41]. Indeed, subsequent large-scale comparative genomics studies of cultured strains revealed extensive recombination between strains of *C. trachomatis*, including loci (i.e. *omp*A gene) that formed the foundation of decades of sero- and genotyping work on this important human pathogen [42, 43]. Another clinically relevant dogma smashed through genomics involves resolution of the ‘peptidoglycan anomaly’, in which chlamydial species, despite being sensitive to beta-lactam antibiotics, were thought to lack peptidoglycan. Genome and transcriptome sequencing respectively revealed the presence of genes encoding the entire pathway for peptidoglycan biosynthesis and assembly [36, 44, 45], and developmental-stage-dependent expression with peptidoglycan genes upregulated during the transition phase between EBs and RBs [45].

These are just two examples of the added value of genomics studies to complement biochemical, cell biological and epidemiological characterization of these intriguing bacteria, and provide further context for the need for development and use of culture-independent methods.

### Chlamydial genomes without culture

Given the value of whole genomes for phylogenetic, epidemiological and biological studies, there is intensified interest in obtaining chlamydial genomes from both clinical and environmental samples. For intracellular bacteria such as *Chlamydia*, *in vitro* culture is usually required to obtain sufficient material for whole genome sequencing (WGS). Due to the laborious nature of cultivating chlamydiae in host cells, the often low numbers of pathogens or presence of inhibitors in clinical samples, the propensity for bacteria to mutate after several passages [46, 47], and the fact many samples are collected into lysis buffer rather than transport media, culture-independent genomic approaches are an attractive alternative gaining recognition and use throughout the chlamydial field.

To overcome the challenges associated with chlamydial culture, groups within the *Chlamydia* field have recently developed several culture-independent genome sequencing methods of sample processing that yield sufficient DNA for WGS, providing unprecedented ability to assemble whole genomes directly from complex or challenging samples (Table 1). These methods can be applied to routine diagnostic samples, and their application not only drastically reduces processing time and cost, but allows for higher throughput and hence greater resolution of phylogenetic analysis.

**Table 1.** Overview of culture-independent genome sequencing methodologies applied to *Chlamydiae* to date CRB, *Chlamydia*-related bacteria; IFU, infection-forming units; IMS, immunomagnetic separation; LPS, lipopolysaccharide; MDA, multiple displacement amplification; WGA, whole genome amplification.

**Culture-independent approach**	**Non-targeted (meta)genome capture**				**Targeted genome capture**		
**Method**	**MDA**	**Depletion-enrichment**	**Cell-sorting-MDA**	**IMS-MDA**		**Sequence capture**	**Multiplexed microdroplet PCR**
Molecular basis of method (also see Fig. 1 for schematic diagram)	Isothermal strand-displacing whole genome amplification of total genomic DNA	Depletion of host methylated DNA coupled with WGA of microbial DNA supernatant by MDA	Fluorescence-assisted cell sorting coupled with WGA of isolated single cells of interest (identified by PCR)	Anti-LPS antibody binding coupled with WGA by MDA		Biotinylated RNA probe hybridization to pathogen genome ‘bait’	Microdroplet PCR amplification of multiple DNA fragments spanning the target genome
Sample type(s) applied to (also see Fig. 2 for method selection)	Swab	Swab, tissue	Environmental samples	Swab stored in transport media		Swab, tissue, urine	Swabs, urine
Sample preparation stage at which to apply method	After total DNA extraction	After total DNA extraction	Initial sample, prior to DNA extraction	Initial sample, prior to DNA extraction		After total DNA extraction	After total DNA extraction
Discovery of novel species?	Yes	Yes	Yes	No		No	No
Detection of multiple strains/co-infection?	Yes	Yes	No	Yes		Yes	Yes
Cost per sample*	$	$	$$$$	$		$$¶	$$$
Speed* (hands on time)	<1 h§	~3–4 h§	~30 min – 2 h§	~5 h§		<1 h	~1–3 h
Sensitivity†	Ct value 25||	~1×10^3^ genome copies per section	315 cells [58]	4 IFU per swab [66]; 1.1×10^5^ genome copies (post-IMS) [65]		~2×10^3^ genome copies per swab [72]; 3.3×10^5^ (urine), 5.5×10^6^ (swab) [69]	Not reported
Specificity‡	99.6 %||	99 % [55]	n/a	Up to 95 % [66]		88 % [74]	99 %
Variant detection?	Yes	Yes	Yes	Yes		Yes	Yes
Throughput	Moderate	Low	Low	High		High	Moderate
Limiting factors(s)/requirement(s)	Relative abundance of microbial and eukaryotic DNA in sample	Relative abundance of microbial and eukaryotic DNA in sample	Cells intact; minimal sample treatment Sample must be cryopreserved Samples in aquatic suspension are preferred	Cells intact; minimal sample treatment Suitable characterized surface-exposed antigen Sample must be preserved in transport media		Reference genome	Reference genome Specific bioinformatics software required
Chlamydial species applied to	*C. trachomatis*	*Ca*. C. sanzinia, *Ca.* C. corallus, *Ca.* Similichlamydia epinephelii	Novel CRB species	*C. trachomatis*		*C. trachomatis*, *C. pecorum*, *C. pneumoniae*, *C. psittaci*	*C. trachomatis*
Reference(s)	[50]	[55–57]	[58]	[64, 65]		[69, 72, 74, 75]	[77]

*Excluding DNA extraction and sequencing; relative cost to each other.

†Lowest amount of chlamydial DNA for 100 % genome coverage.

‡Highest percentage of non-chlamydial reads still allowing 90–100 % chlamydial genome coverage; will differ depending on sequencing platform and degree of multiplexing.

§MDA amplification step not included in time.

||Only 85 % of genome covered at least once.

¶Cost estimate for 1 : 1 bait/sample ratio.

Deep sequencing of metagenomes is another invaluable tool that is particularly useful for discovering new species within their communities. This also addresses the requirement of sequence data for formal proposal and classification of novel chlamydial species, which has previously heavily relied on *in vitro* culture. It is anticipated that the expanding use of these culture-independent metagenomic approaches will provide insight into the unique host–pathogen interactions exhibited by chlamydiae in novel infections for which no culture system exists.

This review thus focuses on recent advances in the development and use of culture-independent approaches for sequencing chlamydial genomes or metagenome-assembled genomes from clinical samples. We review recent studies that have detailed these approaches, explain the molecular basis and compare the utility of each method. We hope this review will provide a starting point when deciding how best to collect and process samples to yield well-covered chlamydial genomes.

## Culture-independent approaches for chlamydial genomics and metagenomics

### Non-targeted metagenomic techniques

**Fig. 1.**
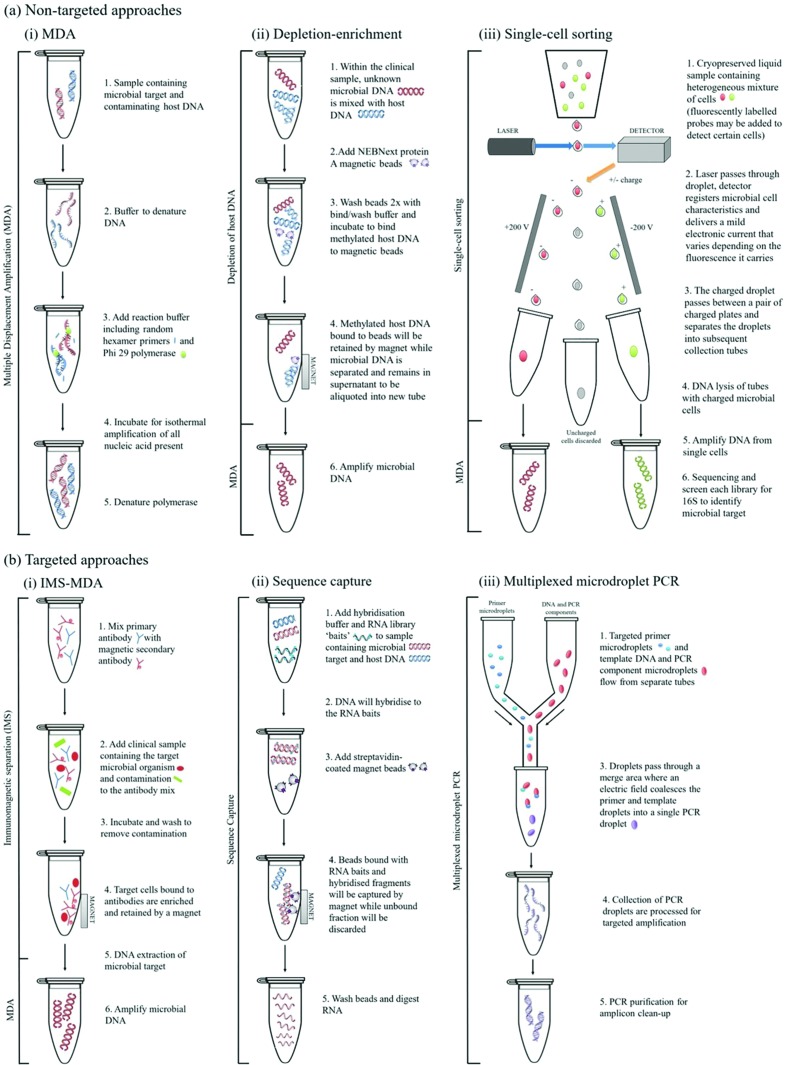
Schematic depiction of culture-independent genome sequencing methods for chlamydiae. (a) Non-targeted approaches for (meta)genome sequencing: (i) MDA, (ii) depletion-enrichment, (iii) cell-sorting-MDA. (b) Targeted genome-sequencing approaches: (i) IMS-MDA, (ii) sequence capture, (iii) multiplexed microdroplet PCR. Refer to Table 1 and Fig. 2 for suitable applications of each method.

Non-targeted (meta)genome capture methods are a useful tool for the isolation and amplification of microbial DNA from mixed clinical samples when the target pathogen is unknown and/or uncultivable. Three non-targeted genome capture techniques have been used for culture-independent sequencing of *Chlamydiae* (Table 1), as depicted in Fig. 1.

#### Multiple displacement amplification

A common method used in both targeted and non-targeted approaches is the use of multiple displacement amplification (MDA), which allows for isothermal, strand-displacing whole genome amplification (WGA) from clinical samples [24, 48, 49] and single cells [15, 17] (Table 1, Fig. 1). The method effectively increases the yield of total or target DNA so that it is sufficient for WGS. Andersson *et al.* reported a single *C. trachomatis* genome from a discarded diagnostic clinical swab (Ct value 25) subject to MDA [50] (Table 1). This yielded low coverage depth over 85 % of the chlamydial chromosome, high coverage of the plasmid, high coverage of several vaginal bacterial species and an over-abundance of human reads. The coverage was only sufficient to call SNPs across 8 % of the chromosome, highlighting a pitfall of sequencing directly from complex clinical samples. The study emphasized the usefulness of WGA for metagenome sequencing and analysis, and to address these issues, MDA has been coupled with depletion methods, described below for both targeted and non-targeted approaches.

#### Depletion-enrichment

A new depletion-enrichment approach has gained popularity in circumstances where the pathogen is either (a) unknown, (b) uncultivable, (c) in low abundance and/or (d) potentially novel. This method, although more expensive than the targeted methods outlined below (Table 1), yields the most information with regard to the entire metagenomic content at the site of infection.

The method relies on separation of non-methylated (i.e. microbial) DNA from methylated (i.e. eukaryotic host) DNA [27], which is then further purified prior to WGA by MDA, thereby enriching complex specimens for prokaryotic DNA (Fig. 1). This approach was used to characterize the genome of a plant pathogen, *Ca.* Liberibacter asiaticus [51], and subsequently the genomes of fish pathogens from sea bream [52–54] (Table 1). One drawback is that sequencing results are heavily dependent on the level and proportions of chlamydial/bacterial DNA in the sample material, and other abundant bacteria may hence mask full genome characterization of the chlamydial species [52, 55]. In some cases, bacterial DNA was further enriched by microdissection of cysts from the gill tissue [52, 53], increasing the specificity of enrichment and subsequent sequencing.

In the chlamydial field, this depletion-enrichment approach has led to a number of advances in our understanding of chlamydial biology. This metagenomic approach allows for investigation of biological characteristics in cases where no *in vitro* culture system has been established, which is the case for a number of *Chlamydia*-related bacteria (CRB) species. Culture-independent genomic methods allowed the first gill-associated CRB to be sequenced and characterized [56], revealing conservation of the chlamydial type three secretion system in early-divergent chlamydiae. This approach also offers insights into chlamydial diversity: two novel *Chlamydia* species were recently discovered in the choana of captive snakes using this method [55, 57] (Table 1). Interestingly, despite near-full length 16S rRNA gene sequence similarity suggesting most of the novel genotypes were *C. pneumoniae* strains, WGS showed that in fact two of the sequences represented novel species, demonstrating the value of WGS to further explore, characterize and classify the ever-expanding diversity of the *Chlamydiales*.

#### Cell-sorting MDA

A third non-targeted metagenome approach for chlamydial genomics is the use of cell-sorting of cryopreserved samples to obtain single cells, differentiated on the basis of fluorescence, size and granularity (Fig. 1) [58, 59]. The DNA from a single cell or pool is then enriched using MDA or other WGA methods [60], to obtain a higher yield of DNA from a single genome. After single cells or pools of cells are obtained by flow cytometry, they are screened using 16S rRNA PCR, or another target gene, to identify the cell or pool of interest. Alternatively, fluorescently labelled antibodies or probes can be used to increase the specificity of sorting.

Cell-sorting MDA (i.e. single-cell genomics) recently enabled characterization of three novel chlamydial genomes from water samples from British Columbia and sediment samples from the North Sea, which revealed a near-complete chlamydial flagellar system [58]. This method offers an advantage over the above depletion-enrichment method in that only the genome of a single species is amplified, rather than the entire microbiome. However, this method has thus far only been applied to liquid samples (i.e. water column samples) in the chlamydial field, and the application to any other sample type (such as a swab) requires minimal treatment into aquatic suspension prior to cryopreservation (Table 1). Despite the observation that genomes recovered by this method are commonly incomplete or highly fragmented [58, 61], the information gained regarding metabolic adaptations is invaluable when cultivation is a challenge.

### Targeted capture methods

Targeted capture methods rely on prior knowledge of the target bacteria, e.g. a reference genome or well-tested antibody (Table 1). Two targeted techniques have been used for culture-independent genome sequencing of *Chlamydiae*, and both ‘capture’ either the genomic DNA or intact cells of the desired microbial species using species-specific molecular targets (Fig. 1).

#### Immunomagnetic separation – multiple displacement amplification

Building on previous assays employing immunomagnetic separation (IMS) for diagnostic purposes such as *Listeria* detection in food sources [62], and *C. trachomatis* in urine samples [63], chlamydial IMS-MDA uses primary mouse IgG antibodies directed at the chlamydial lipopolysaccharide (LPS), an antigen found on the chlamydial cell surface. A secondary IgG conjugated to magnetic beads then binds intact chlamydial EBs (Fig. 1) [64–66]. Host DNA is removed by DNase treatment prior to DNA extraction from the bound EBs, MDA and sequencing.

The IMS approach alone was found to be highly specific for *C. trachomatis*, with only contaminating reads from human DNA, rather than other microbial DNA seen in non-targeted approaches. However, results were further enhanced by the addition of the MDA step: Seth-Smith *et al.* noted that clinical swabs for *C. trachomatis* rarely carry sufficient quantities of target bacterial DNA for genome sequencing, and MDA had previously been successfully used to obtain complete bacterial genome coverage from mixed samples [50, 67, 68], despite very low starting concentrations of target DNA material required for sequencing [65].

Putman *et al*. then applied the IMS-MDA method to ten *C. trachomatis*-positive endo-cervical swabs. While lower inclusion-forming units (IFU) loosely correlated with more unsolved bases, the authors showed this method was highly sensitive, with whole genomes obtained from samples containing as little as 4 IFU per swab prior to enrichment (Table 1) [64]. This study had two main findings that may have been overlooked had these samples been prepared and sequenced according to traditional methods (culture followed by EB purification). In one sample, unresolved bases reflected a within-host clonal population, and in another, clonal variation at two nucleotide positions separated by 150 000 bp suggested active recombination in response to selective pressures not present *in vitro* (based on culture and sequencing of the same sample).

#### Sequence capture

*C. trachomatis* has also been the subject of another culture-independent approach which uses biotinylated RNA probes (‘baits’) to hybridize chlamydial DNA away from a complex DNA mixture to ‘capture’ the chlamydial DNA (Fig. 1) [69]; this method has successfully yielded viral genomes from mixed samples [70]. The 120-mer custom commercially synthesized RNA bait sets are designed to span the entire chromosome and hybridization occurs after library preparation. Like IMS-MDA, this method uses magnetic separation, in this instance using streptavidin-coated beads to separate the hybridized DNA, prior to genome sequencing. This method has been applied to DNA extracted from both swab and urine samples, with a higher sensitivity obtained with urine samples, potentially due to lower background DNA (Table 1). Over 95 % of the length of the chromosome was covered by as little as ~1 % of the reads in samples with as little as 1×10^4^ chlamydial genome copies. It is worth noting that in this case clinical samples required sequencing twice to obtain the same coverage as cultured samples [69], and this is highly dependent on the sequencing platform and degree of multiplexing. More recently, the phylogenomic relationships between globally diverse *C. trachomatis* strains were resolved by genome sequencing following culture, IMS-MDA and sequence capture [71].

Sequence capture has also been applied to the culture-independent genomic analyses of veterinary chlamydiae in clinical specimens. *Chlamydia pecorum* genomic studies using the probe–bait hybridization method have revealed that, as previously suspected, livestock and koalas may be colonized with more than one strain of *C. pecorum* at the same anatomical site. These whole chlamydial genomes, obtained from dry koala ocular and urogenital swabs and bovine joints and ocular swabs, could be separated from each other based on abundance, and were phylogenetically distinct, separated by over 6 000 SNPs [72, 73]. The presence of ‘minor strains’ in clades dominated by Australian livestock supports ongoing exposure and potential cross-transmission of livestock strains into koalas from sheep and cattle.

While these methods have proven valuable for analysis of clinical specimens, this approach has also been used advantageously to analyse chlamydial isolates (a) following just a few passages of chlamydial cell culture [74] and (b) that were no longer culture viable [75]. These again yielded full coverage of the target genome despite some read sets only containing 12 % chlamydial reads [74]. While the methods used in these examples were not strictly culture-independent, processing time was reduced substantially by extracting DNA directly from culture material rather than carrying out EB purification from host cells [76].

#### Multiplexed microdroplet PCR

A final targeted approach that has so far only been used to amplify a 100 kb region of the *C. trachomatis* genome is multiplexed microdroplet PCR, which uses 500 primer pairs to generate overlapping 1–1.3 kbp amplicons spanning the selected region [77] (Table 1). A primer microdroplet library is prepared separate to the DNA and PCR component droplets. The two are then emulsified prior to PCR amplification (Fig. 1) [78]. Like IMS-MDA and bait–probe hybridization, this method is heavily dependent on a reference genome from which to design primer targets. It also requires very specific downstream bioinformatics methods which the authors developed to address this [77]. The microdroplet PCR sequencing approach is a culture-independent sequencing method intermediate between multi-gene sequencing and WGS that could be scaled up to cover a complete genome. Its development has been shown to be valuable for *C. trachomatis* phylogenetic and epidemiological studies, and the method is capable of discriminating between strains in a sample [77]. Importantly, the approach successfully yields sequences from discarded clinical samples (swabs and urine), although areas of recombination were hard to resolve.

### Method selection

**Fig. 2.**
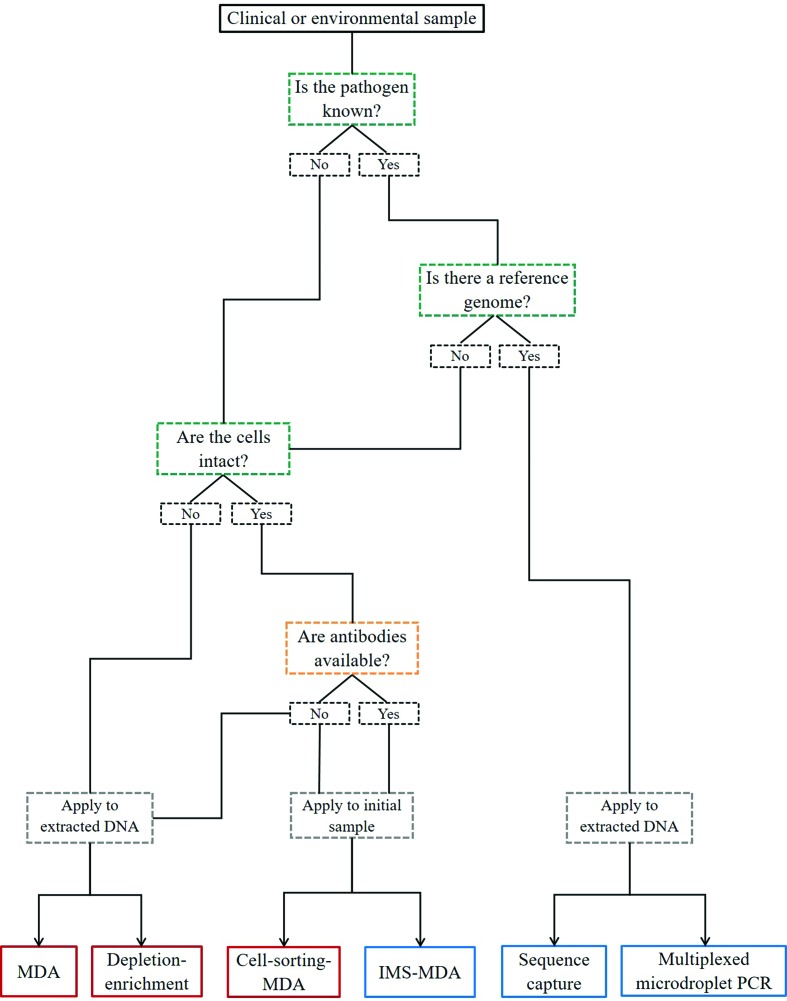
Application of culture-independent genome sequencing methods to chlamydiae. Decision tree for choosing culture-independent genome sequencing method for chlamydiae. Green boxes denote primary criteria for method selection, with downstream key decision points boxed in orange and sample stage application boxed in grey. Non-targeted approaches are boxed in red and targeted approaches in blue. Refer to Fig. 1 for an overview of each method.

This review has summarized the key culture-independent approaches for chlamydial genome and metagenome sequencing. As outlined in Table 1 and Fig. 1, each approach has varying technical requirements for (a) isolation of microbial or chlamydial cells or DNA from mixed samples and/or (b) enrichment of the microbial or chlamydial DNA in preparation for sequencing and analysis. We also present simple selection criteria for choosing the appropriate DNA preparation method and culture-independent genomic approach for your samples and research questions (Fig. 2).

Throughout this review, we have categorized these methods as targeted and non-targeted approaches, which is determined primarily by whether the target bacterium is known (Fig. 2). The non-targeted approaches are not exclusive to samples for which the pathogen is known, but are more expensive and of lower throughput than the targeted approaches. Method selection is also dictated largely by the sample type and the way it has been collected or stored and if the bacterium of interest has a reference genome from which to design species-specific capture tools (Fig. 2). This makes the non-targeted methods ideal for pathogen discovery, while the targeted methods are best suited to diagnostics and epidemiology where lower cost, higher throughput and high sensitivity are desirable (Table 1).

As an example, if we have a discarded diagnostic *C. trachomatis*-positive urogenital swab in lysis buffer, we could apply MDA, depletion-enrichment, sequence capture or multiplexed microdroplet PCR (Fig. 2). Of these, we would probably opt for sequence capture, as we have a reference genome from which to design baits (Fig. 1), and it is fairly cheap, of high throughput, sensitive and specific (Table 1). However, if the swab were collected and stored in appropriate storage media to preserve viability of intact cells, we may perform IMS-MDA instead (Fig. 1). On the other hand, if we have a *Chlamydiales*-positive tissue sample but no species-level identification or it represents a putative novel species, we would either choose MDA or depletion-enrichment (Fig. 2). In this case, depletion-enrichment, although slower and more costly, would probably yield higher coverage of the target genome (Table 1).

## Final remarks and future directions

Although chlamydial genomics has come a long way in only 20 years, there are several avenues yet to be explored. Our knowledge is currently limited only by the sample types to which culture-independent genome sequencing has been applied in the chlamydial field to date (Table 1), and there is scope for some methods to be applied more widely. It is also important to note that no studies have directly tested these methods on the same sample, and such a study would clarify their advantages and limitations. Furthermore, the depth of sequencing was not consistent between the studies reviewed here, which is a factor that would affect sensitivity and specificity (Table 1), and warrants further comparison.

To date, the targeted approaches described here have only been applied to *C. trachomatis*, *C. pecorum*, *C. psittaci* and *C. pneumoniae.* Reference genomes now exist for all *Chlamydia* species, so targeted approaches to enable high-throughput sample processing and sequencing of other species is plausible. As many CRB genomes are also now sequenced and complemented by proteomic and transcriptomic studies, these approaches could also be extended into CRBs [79]. Importantly, these approaches are capable of discerning two strains in the same sample, a feat that would probably not be achieved if *in vitro* culture was undertaken prior to sequencing. A limitation to this method is the bias against detecting novel genes or extrachromosomal elements, as was the case for *C. pecorum*, for which the chlamydial plasmid sequence was only resolved due to the sequence similarity between chromosomal and plasmid genes captured using the RNA baits [80].

Non-targeted culture-independent genomic approaches will be fundamental in identifying novel species in novel hosts or environments and hence in further characterizing chlamydial diversity. Coverage uniformity appears to be a common limitation for MDA [13]. MDA could be combined with other depletion or isolation methods, such as laser capture microdissection of inclusions from fixed tissue or cells, although fixing samples can result in fragmented DNA, which is not ideal for WGS. Single-cell genomics is very much in its infancy in the *Chlamydia* field but holds great promise as it is highly specific and sensitive (Table 1): it is yet to be applied to swab, urine or tissue samples but this could be developed in the future.

We did not delve into the downstream bioinformatics methods required to assemble and analyse genomes generated from these culture-independent methods, as we feel this would warrant a separate review. However, it is worth noting that different approaches may require different bioinformatics methods, of which there are a plethora.

Sequencing and characterization of chlamydial genomes has revolutionized our knowledge of chlamydial biology, diversity and evolution. The advent of chlamydial genomics was stunted by the requirement for *in vitro* culture, but chlamydial culture-independent genome sequencing will contribute more, diverse genomic data to our knowledge of this unique, expanding phylum.

## Data bibliography

1. All data referenced in this review have been properly cited and is detailed in the reference list.
